# Novel High Intensity Focused Ultrasound in Debriding Ti Dental Implant-Attached *Streptococcus mutans* Biofilms

**DOI:** 10.1016/j.identj.2025.103905

**Published:** 2025-09-18

**Authors:** Minh Dien Tran, Alysia Hubbard, Catherine A. Rinaldi, Hien Ngo, Sheetal Maria Rajan, Amr Fawzy

**Affiliations:** a Biomaterials and Dental Technology Research Team, UWA Dental School, the University of Western Australia, WA, Australia; b Centre for Microscopy, Characterisation and Analysis, the University of Western Australia, WA, Australia

**Keywords:** Peri-implantitis, Biofilms, *Streptococcus mutans*, HIFU, Ultrasound, Debridement

## Abstract

**Introduction and aims:**

Effective management of PI depends on removing the colonised microbial biofilms to preserve osseointegration, yet thorough debridement remains difficult. HIFU offers a novel, non-invasive approach that uses contact-free acoustic energy while minimising aerosol and debris. This in vitro study evaluates the effectiveness of HIFU in removing *Streptococcus mutans* biofilms from titanium implants. Biofilm reduction was visualised via DCLSM and quantified using FCM.

**Methods:**

Two Ti dental implants were characterised via SEM. *S. mutans* biofilms were grown over 10 days on the coronal 5mm section of another 18 implants. Implants in the test group (n = 6) were treated with optimised HIFU through a glass beaker in water-based medium for 2 minutes. Residual biofilms were stained and visualised under DCLSM at 4×, 10×, and 20× magnifications and quantified using FCM for live/dead bacterial images and counts. The data from the test and the control samples (untreated and Airflow® treated) were subjected to ANOVA followed by post-hoc Tukey’s test to determine the statistical differences between the groups.

**Results:**

DCLSM Z-stacks using a 10× objective and 512-pixel resolution yielded the optimal balance between image clarity and field coverage. HIFU-treated implants showed scattered bacterial clusters with surrounding biofilm-free zones, while Airflow®-treated samples retained a thin, continuous biofilm primarily along thread walls and root junctions. Both Airflow® and HIFU significantly reduced total bacterial loads compared to untreated controls (*p* < .05). (Airflow: 40,021 ± 17,253; HIFU: 254,000 ± 124,000; control: 731,000 ± 211,000).

**Conclusion and clinical relevance:**

HIFU acoustic waves were uniquely able to penetrate a solid (glass) barrier and create localised biofilm-free regions. HIFU shows potential as a physical method for biofilm disruption; however, further studies - including multispecies, *in vivo,* and pre-clinical models - are required to evaluate its clinical applicability.

## Introduction

PM and PI are the 2 conditions categorised under peri-implant diseases (PDs).^1^ Both are inflammatory disorders triggered by biofilm accumulation, impacting the soft tissues and, in the case of PI, the underlying bone supporting dental implants. PM is defined by inflammation limited to the soft tissue surrounding the implant, without any accompanying bone loss, and is considered a reversible condition.^2^ However, if not addressed promptly, PM may advance to PI—a more severe, irreversible form involving both mucosal inflammation and progressive bone destruction.^1^ This condition poses a major challenge in contemporary dentistry due to its high prevalence, destructive consequences, and the growing number of implants placed worldwide.^3^^,^^4^

PI affects around 20% of dental implants after 5 years, and this prevalence tends to increase the longer the implants remain in service.^5^, ^6^, ^7^, ^8^ As of 2023, the annual number of dental implants placed globally was estimated at 12 to 15 million, and the global market for dental implants and prosthetic devices is expected to grow at an annual rate of around 7.5%, reaching an estimated value of USD 16 billion by the year 2029.^9^ The growing number of dental implants placed worldwide, combined with the high prevalence of peri-implantitis, contributes to a substantial economic burden associated with the management of this condition. According to Grand View Research, the global market size of PI was estimated USD 1.04 billion in 2025 and projected to grow at 9.1% annually.^10^ Amid this escalating health and economic impact, current strategies for prevention and treatment remain inadequate, as PI persists with high prevalence and recurrence rates, often culminating in implant loss along with surrounding bone and soft tissue destruction.^11^^,^^12^ Although there is a number of contributing factors, microbial biofilms on implant surfaces are widely recognised as a primary etiological factor in PI,^1^^,^^13^, ^14^, ^15^ making the eradication of these complex microbial communities essential for effective management. The microbiology of PI is intricate, and a definitive bacterial profile has yet to be established.^16^, ^17^, ^18^ Systematic review by Pérez-Chaparro^19^ identified microorganisms from 6 bacterial phyla, 17 genera, 23 species, and 2 viral genera linked to PI. Among these, anaerobic bacteria such as *Aggregatibacter actinomycetemcomitans, Prevotella intermedia, Porphyromonas gingivalis, Treponema denticola, Tannerella forsythia*, and *Campylobacter rectus* have been particularly implicated. Despite the lack of direct evidence associating *S. mutans* with PI,^19^ this bacterium is routinely found in subgingival biofilms from individuals with healthy gingivae and patients with periodontal disease.^20^ These facultative anaerobes exhibit a rapid exponential growth rate of 0.24 per hour^21^ and play a critical role as early colonisers in the initial stages of biofilm development.^22^

Owing to the intrinsic microbial resistance of multispecies biofilm communities,^23^^,^^11^^,^^24^, ^25^, ^26^, ^27^, ^28^ physical debridement methods have been extensively studied.^29^, ^30^, ^31^, ^32^, ^33^ Regardless of the differences in treatment outcomes between surgical and non-surgical approaches,^34^ implant surface decontamination remains a fundamental component of the disease management^34^^,^^35^ and prevention.^36^, ^37^, ^38^ Nonetheless, this fundamental therapeutic approach has encountered numerous challenges.^12^^,^^39^ The effectiveness of managing peri-implantitis using manual tools (made from materials like titanium, plastic, Teflon, or carbon fiber), mechanical instruments (such as air-abrasive systems, rubber polishing cups, and sonic scalers), and various lasers (including Er, Cr:YSGG, and Er:YAG) have been reported but remains inconclusive. This is primarily due to the limited number of clinically controlled trials and the considerable variability in study protocols.^16^^,^^39^ Airflow® is an air abrasive system that has been an established technology in clinical practice for debriding implant biofilms both supra- and subgingivally. Although its restricted efficacy has been reported,^40^, ^41^, ^42^, ^43^, ^44^ the adverse effects range from aerosol generation, residual powder remaining on the implant surface, subcutaneous emphysema,^45^ epithelial desquamation^46^ to life-threatening pneumocephalus.^47^ Another important observation is the consistent pattern seen across these reported methods: they deliver energy in a pulsatile fashion. This involves localised, intermittent bursts applied incrementally to the target area, which are likely to result in microscopic regions being left untreated

To address these existing scientific and clinical limitations, High-Intensity Focused Ultrasound (HIFU) emerges as an innovative approach for targeting biofilms on dental implant surfaces, with potential applications in both the prevention and management of PI. Unlike existing techniques that primarily deliver pulsed energy, this soundwave presents a distinct set of advantages: it is non-radiative, non-ionising, operates without auxiliary materials, and produces minimal aerosol. Furthermore, it transmits a clean, continuous acoustic wave to the hard-to-reach targets with accuracy and precision without requiring direct contact between the ultrasound source and the treatment site.^48^, ^49^, ^50^

Although this acoustic technology has a well-established history dating back to 1927^51^ and has been widely utilised in clinical medicine,^50^^,^^52^ its dental applications remain limited and largely restricted to the research setting. Building on the positive findings from previous microbiology-related studies^49^^,^^53^ involving *S. mutans* biofilms adhered to Ti discs with surface roughness resembling that of dental implants, we hypothesised that HIFU could effectively remove bacterial biofilms from Ti dental implants.

A combination of DCLSM imaging and FCM quantification was employed to analyse the anti-biofilm effect of these 2 techniques. This dual fluorescence and reflection CLSM technique was first used by Baschong et al. in 2001^54^ to image Ti implant-tissue interfaces. While not commonly applied in dental implant research, this technique offers valuable insights into the visualisation of adherent biofilms and their spatial distribution relative to the Ti surface topography.^49^^,^^55^ Although FCM has been widely studied in medical research,^56^ its application to bacterial biofilms associated with dental implants remains limited. This innovative combination of 2 biofilm analysis techniques was validated in analysing *S. mutans* biofilms on titanium surfaces^49^ and was employed again in this study to test the hypothesis.

## Materials and methods

### Study design

**Fig. 1:**
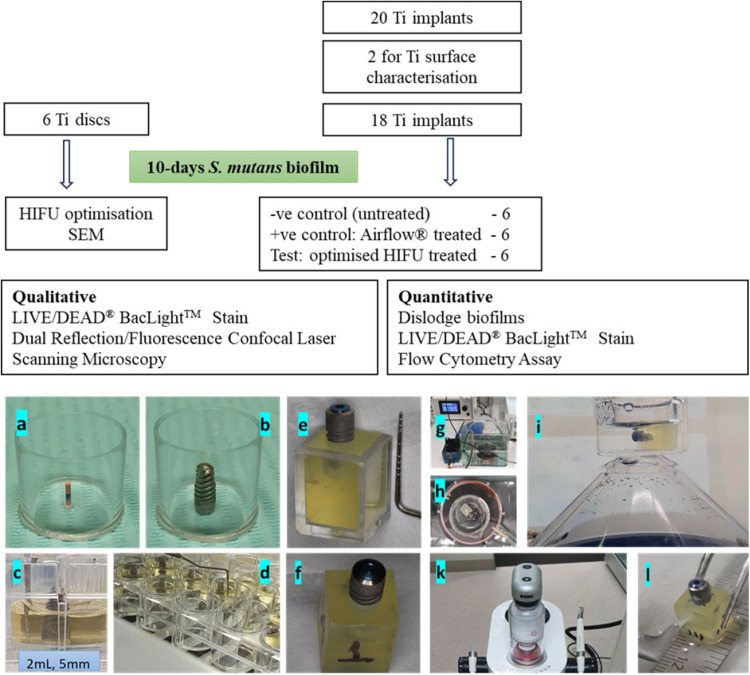
**Above**: Flowchart outlining the study design using 6 Ti discs for HIFU optimisation, 2 Ti implants for surface characterisation, and 18 Ti implants for biofilm debridement experiments. **Below**: Graphic illustrations showing biofilm culturing on titanium implants (**A-F**) and the experimental setups for biofilm debridement using HIFU (**G-I**) and Airflow® (**K-L**). The implant–biofilm assemblies were fully isolated from the HIFU transducer by the base of a 1 mm-thick glass beaker and an additional 3 mm of PBS.

This study utilised 20 titanium dental implants (REF: DCT4009, 4 mm in diameter and 9 mm in length) manufactured by Southern Implants Pty Ltd., Irene, South Africa. The implant surfaces were roughened through alumina-grit blasting. In addition, to optimise HIFU intensity, six Ti discs (10 mm in diameter, 78.54 mm^2^, 2 mm in thickness) produced by the same manufacturer as the implants were used. The testing side of the discs, where biofilm growth took place, was roughened while the remaining non-testing area of the disc was polished to minimise bacterial adhesion. Study design is summarised in Figure 1.

### Ti surface characterisation

To qualitatively characterise the testing surface, a pair of implants was affixed onto aluminium SEM stubs using Cu tape, carbon-coated (∼20 nm) (Polaron SC7640, Quorum Technologies Ltd, U.K), and examined using Scanning Electron Microscopy (SEM) with a Zeiss 1555 VP-FE SEM. SEM images of the crest module (collar, neck) (Figure 5) and adjacent body, where biofilms were expected to be grown, were taken at 100x, 500x, and x5,000 magnification. Implant thread design, dimension, and surface roughness of the implant section used in the study were recorded following Misch’s (Misch 2008) implant nomenclature.^57^

### *Streptococcus mutans* biofilm culture

To facilitate bacterial biofilm formation on the implants, this study utilised sterile 24-well culture plates for sample preparation. To secure the implants in a vertical position inside each well, a 5mm long X3 PROTAPER NEXT gutta-percha (GP) endodontic point cut-outs (Dentsply Sirona) were fixated vertically at the centre of the wells with a thin layer of adhesive (Ethyl-2-cyanacrylate, Sekundenkleber) (Figure 1, A and B). The prepared wells were immersed in 80% ethanol for 5 minutes for disinfection. The ethanol was gently shaken off, and the wells were left to dry under ultraviolet light inside a microbiological workstation (Cruma Australia Laminar Flow Cabinet) for 20 minutes.

Glycerol stock of *S. mutans* from (ATCC 700610) was cultured in 5 mL of Brain Heart Infusion (BHI) (Sigma-Aldrich) medium. This broth was streaked on blood agar and incubate at 37 °C for 48 hours. A single colony was grown overnight in BHI broth supplemented with 1% sucrose (Sigma-Aldrich) at 37°C and adjusted to a concentration of 5×10^6^ colony-forming units (CFU) per mL (optical density at 600 nm = 0.5) (McFarland Densitometer – Fisher Biotec).^49^

To grow the biofilms [modified protocol from Lemos et al.^58^ and ATCC bacteriology culture guide], the implants were placed vertically in the prepared 24-well plate using a GP post at the holding mechanism (Figure 1, A and B). The well was then filled with 2 mL of adjusted bacterial suspension. This volume of the suspension covered 5mm of the implant collar and nearby body, while the remaining 4mm at the apex of the implant stayed dry, minimising bacterial adherence and growth. This growth-limited apical section of the implants allowed the instrumentation of the implants without disturbing the biofilms. The plate was incubated at 37 °C in an orbital shaker at 50 rpm, and the medium was replenished every 2 days (Figure 1, Fig. 1, Fig. 1).

On day 10th, the implants were picked with a pair of sterile tweezers, gently washed in 4 mL of PBS (x4) to remove loosely attached bacteria and debris, then placed in 2 mL of fresh PBS in a 24-well plate, ready for experimentation. The implants were randomly divided into 3 groups of equal numbers (n = 6), namely untreated control (-ve control), Airflow®-treated (+ve control), and HIFU-treated (test) using simple randomisation in Microsoft Excel (RAND function). The -ve control samples were not subjected to biofilm debridement methods, while +ve implants were treated with the air-powered abrasive of Airflow®, and the tests with high-intensity focused ultrasound (HIFU).

Similar protocol was used to grow biofilms on Ti discs for HIFU optimisation, for Ti discs. Ti discs were positioned on their non-testing (polished) surface at the bottom of the wells.

The discs were divided into 3 groups of 2, namely control (untreated), 20 W (treated with 20 W HIFU intensity) and 30 W (treated with 30 W HIFU intensity).

### Implant – biofilm preparation

The biofilm-coated implants were placed into sterile resin mounting blocks with a square column geometry measuring 9 × 9 × 11 mm following the implant manufacturer’s osteotomy protocol, inserted to a depth of 4 mm, leaving 5 mm of the coronal portion with attached biofilms exposed. These implant mounting blocks were fabricated through an injection-moulding process, utilising the visible light-cured resin Triad® Gel (Dentsply Sirona), which was injected into a square plastic column mold (9 × 9 × 11 mm) derived from the lower section of a Kerr Dental (Brea) gutta-percha cartridge. The blocks were subsequently cleaned and sterilised in accordance with standard dental practice sterilisation protocols. Care was taken to avoid any contact with the biofilms during the implant placement process. Loose biofilm fragments and debris resulting from implantation were gently rinsed away using phosphate-buffered saline (PBS). The 4 sides of each mounting block were designated as quadrants I, II, III, and IV, representing the peripheral regions surrounding the implant. Cover screws were placed to close the screw access channels (Figure 1, Fig. 1, Fig. 1).

### HIFU setup

A HIFU transducer (H-115, S/N: 046, Sonic Concept), with 64.00 mm in diameter, 41.16 mm focal length and 6.35 mm focal width was placed at the bottom of a rectangular container (270 × 143 × 120 mm dimension) filled with 2 L of distilled water. The container consisted of a desktop aquarium tank constructed from 4-mm-thick plexiglass bonded with aquarium-grade silicone, which functioned as the HIFU chamber. Prior to use, it was disinfected with 80% ethanol and subsequently exposed to ultraviolet light for 20 minutes within a microbiological laminar flow cabinet (Cruma Australia) for additional decontamination. The transducer was connected to a TPO-200-09 version 5.01 HIFU generator (Sonic Concept) through a XDR094-014 fundamental RF impedance matching network at 250 kHz frequency and continuous mode. This generator was a plug-and-play unit with a built-in RF power amplifier and sinusoidal waveform generator, enabling direct transducer drive with adjustable output parameters between 0 and 33 W (Figure 1, G).^49^^,^^59^^,^^60^

**Fig. 2:**
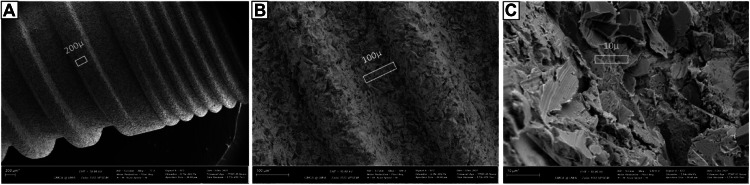
SEM images of the region of the implant collar and adjacent body. (A): spiral thread design with steep thread angles was observed in the collar region, while square design was seen in the body. (B): the dimensions of thread features in the collar; and (C) the roughness of the implant.

The mounted implant was placed horizontally at the bottom of a 25 mL glass beaker filled with 10 mL of PBS. The beaker was positioned on top of the transducer at the focal point. The targeted biofilms were positioned about 4 mm from the HIFU focal point, comprising 1 mm of glass beaker base and 3 mm of PBS solution (Figure 2, I). Each quadrant of the implant was exposed to HIFU beams for 30 seconds, bringing the total HIFU exposure time to 2 minutes. HIFU-treated implant was removed from the mounting blocks, gently washed in 4 mL of PBS (x4), and stored in 2 mL of fresh PBS.

### HIFU optimisation

Ti discs were HIFU-treated according to their groups (control, 20 W, and 30 W). The samples were gently washed 4 times in 2 mL of PBS to remove debris, fixed in 2 mL of 4% formaldehyde solution for 30 minutes, washed with PBS and left dry. The samples were affixed onto aluminium SEM stubs, carbon-coated (∼20 nm) (Polaron SC7640, Quorum Technologies Ltd), and examined using Scanning Electron Microscopy with a Zeiss 1555 VP-FE SEM at 5000 magnification.

### Airflow setup

For the positive control implants, An Airflow unit (EMS 2019 model – KU08654) - which delivered a grit blasting jet mix of water, air, and Airflow Plus powder containing 14 µm erythritol particles (> 95%), chlorhexidine diacetate (0.3%). The implant biofilms were debrided following EMS recommended instructions of 100% water, 60% pressure (2.4 Bar), and the nozzle was kept at right angle and 4mm from the implant surface (using a modified Duratech Precision TH-1989 PCB holder) for 30 seconds to each implant quadrant (Figure 1, Fig. 1, Fig. 1).

The implant was then removed from the mounting blocks, placed in 24 microwell plate filled with 2 mL of PBS for 30s to dilute the residual powder, removed and dipped 4 times in another 4 mL PBS. The implants were then stored in 2mL of PBS solution and were ready for staining within 2 hours.

### Staining and fixation

To prepare the samples for CLSM and SEM, the implant was stained with LIVE/DEAD BacLight (Invitrogen – ThermoFisher Scientific) following the manufacturer’s instructions - 1.5 µL of propidium iodide and 1.5 µL of SYTO9 in 2 mL of PBS for 15 minutes at 37 degrees Celsius. The implant was then removed, gently dipped 4 times in 2 mL of PBS to remove excess stain, fixed in 2 mL of 4% formaldehyde solution for 30 min, and washed with PBS. The stained biofilms were mounted on glass cover slip (0.17 mm thick) and the images were acquired using a Nikon A1 RMP confocal microscope.

### Dual reflection/fluorescence CLSM protocol

To capture the microscopic 3-dimensional images of the Ti implant surface and its attached biofilms, a CLSM technique combining 2 different imaging modes of fluorescence and reflection was performed. This technique was aimed at displaying not only the fluorescent biofilms but also the metallic and highly reflective Ti implant surface to which the biofilms attached.

The samples were imaged on a Nikon A1 RMP confocal microscope. Four sites of each sample (Ti disc or Ti implant) were selected randomly on each quadrant, where Z-stack images were taken. For the control samples, the "top" and "bottom" of the Z stacks were determined based on when the fluorescent-stained bacteria first became visible and then disappeared, respectively, under optical excitation using the recommended 488 nm and 561 nm laser wavelengths. For both +ve control and test samples, the reduced number of stained bacteria and the corresponding decrease in fluorescence emission necessitated the use of reflection mode with a 405 nm laser beam to determine the Z-stack height.

The 4x (NA 0.2), 10x (NA 0.45), and 20x (NA 0.75) objective lenses, 512-pixel and 1024-pixel sizes and the default Z step size were the parameters used throughout the imaging process.

Once the parameters of Z stack had been set up, 2 consecutive Z – stacks were taken on the same region to capture and reconstruct the tri-dimensional morphology of the Ti implant surface and the attached biofilms. For the first Z stack, fluorescent method using 488 nm and 561 nm laser beams (filters 525/50 and 595/50 bandpass filters respectively) was employed to capture the live (SYTO9 stained) and dead (PI stained) bacteria respectively. The stack was then repeated using the reflection technique with 405 nm laser beam to illuminate the surface, and reflected light collected between 405 and 750 nm wavelength range. The 2 Z stacks were then merged using NIS software.

For each site of the sample, 2 sets of 2 Z stacks were collected. A lower magnification data set was first collected using 4x objective lens showing the larger area followed by a higher magnification from 10x and 20x objective lenses focusing on a smaller section of the biofilms showing their distribution, characteristics, and proportion of live and dead bacteria. The pixel size of 512 and step size (15 microns for 4x; 3 microns for 20x objectives) were applied.

The images were processed using NIS-Element AR software by projecting full-view snapshots (8-bit GRB) of all channels, which were then copied into Microsoft PowerPoint (Version 2405) for presentation without any modification in colour, sharpness, or contrast. After analysing images acquired at various magnifications, those captured using the 10× objective lens was selected for presentation in the manuscript, as they were able to display both the attached biofilms and morphological features of the Ti implant substrates.

### Flow cytometry assay protocol

To remove the residual biofilms remained attached to implant surface after treatments, the implants were placed in 5 mL flat-bottom tubes filled with 2 mL of PBS and placed in an ultrasonic bath serving as a sonicator (L&R SweepZone Technology) for 30 minutes. The tubes were then shaken using a vortex mixer (ISG Vortex mixer model 153-010 (International Scientific Group) for 2 seconds.^49^

For bacterial counts using FCM**,** the 2 mL suspension was centrifuged at 4000 rpm for 10 minutes, the supernatant was removed and resuspended in 2 mL of sodium chloride 0.9%. After vortex mixing, 100 µL of this bacterial suspension was stained with propidium iodide (PI) and SYTO9 from LIVE/DEAD BacLight Bacterial Viability and Counting Kit (Thermo Fisher Scientific) following manufacturer’s instructions. Briefly, 1.5 µL of propidium iodide (from 200 µL of a 20mM solution), 1.5 µL of SYTO9 (from 200 µL of a 3.34 mM solution), and 10 µL microspheres (6 µm in diameter, concentration of 1.0 × 10^8^ beads/mL) were diluted to a final volume of 1000 µL of stained bacterial suspension for measurement. This 1000 µL suspension had 30 µM concentration of PI (1.81 × 10^16^ molecules in total), 5.01 µM concentration of SYTO9 (3.02 × 10^15^ molecules in total), and 1×10^6^ microspheres. Four single-color controls were prepared and the results from these controls were used for voltage calibration and gating. Samples were analysed on a BD LSR-Fortessa SORP cytometer and BD FACSDiva software (BD Biosciences), and fluorescent signals were measured on green fluorescence channel ([488] nm laser with 530/30 band pass filter) and red fluorescence channel ([561] nm laser with 610/20 band pass filter). Forward scatter, side scatter and fluorescence data were recorded for 5 minutes per sample using a medium flow (250 µL in total). The files were exported in FCS 3.1 (flow cytometry standard) format and analysed using FlowJo v10 (BD Biosciences) software. The sample quality was ensured by checking the FSC versus time plot, and that 1×10^6^ microspheres were collected from each 1mL sample, if not sample data was discarded. Four single-color controls were used for gating, which differentiated 3 populations SYTO9 stained (live), PI and SYTO9 stained (dead), and total bacteria. The numerical counts of these 3 populations were extracted and used for statistical analysis.

### Statistical analysis

Data from FCM quantitative measurements were processed using Microsoft Excel software version 2410 (Build 18129.20116). All data are presented as the mean ± standard deviation. ANOVA followed by post-hoc Tukey’s test was performed to determine the statistical differences among the groups at the alpha level (*p* < .05).

## Results

### Ti surface characterisation

SEM images of the implant collar and adjacent body were presented in Figure 2, showing the spiral thread design with a steep thread angle in the collar region. This thread design appeared square in the body. The implant collar was measured 3mm in height with 120 µ thread roots and 80 µ in thread depth and crest. The dimensions of the thread depth, root, and crest increased more than doubled in the implant body. At 5000 magnification, the implant surface displayed a markedly irregular topography, defined by sharp, prominent peaks and deep valleys. It appeared coarse and non-uniform, featuring notable microstructural disruptions and a rugged surface profile.

### HIFU optimisation

**Fig. 3:**
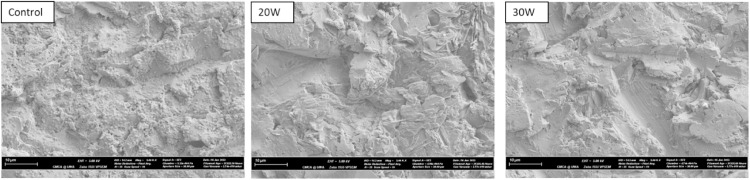
SEM images of biofilms attached to flat Ti surfaces. Among 3 groups, the 30 W HIFU treatment showed the highest level of biofilm removal and was selected for the debridement of Ti implant-attached *S. mutans* biofilms.

SEM images of *S. mutans* biofilms attached to flat Ti surfaces were presented in Figure 3. With 20 W HIFU treatment, the Ti surface showed a tremendous reduction in the bacterial presence, however, a significant amount of distorted biofilm matrix was still present, obscuring the true topography of the Ti surface. The residual matrix was barely observed in the disc treated with 30 W HIFU, making this parameter the choice for implant-attached biofilm debridement.

### Qualitative results - CLSM images

**Fig. 4:**
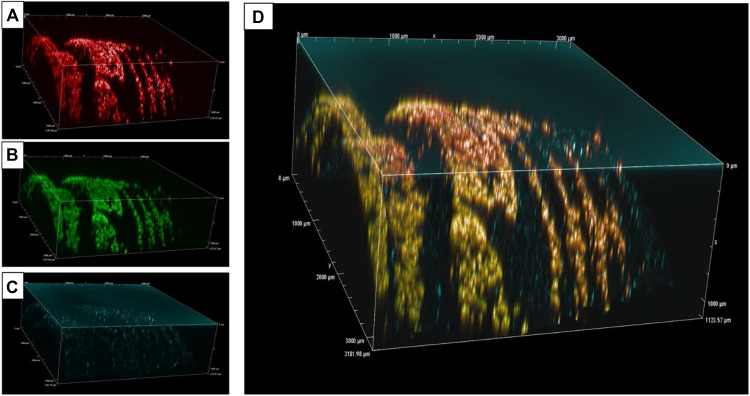
CLSM Z-stack images of *S. mutans* biofilms attached to Ti implants were captured using a 512-pixel resolution with 4× objective lenses**.** The left column displays individual channels acquired using: a 561 nm laser for propidium iodide (PI)-stained dead bacteria (A), a 488 nm laser for SYTO9-stained live bacteria (B) in fluorescence mode, and a 405 nm laser for the titanium implant surface (C) in reflection mode. The right column (D) presents merged images combining the fluorescence and reflection channels. These images failed to adequately reveal details of either the implant surface or the attached biofilms.

**Fig. 5:**
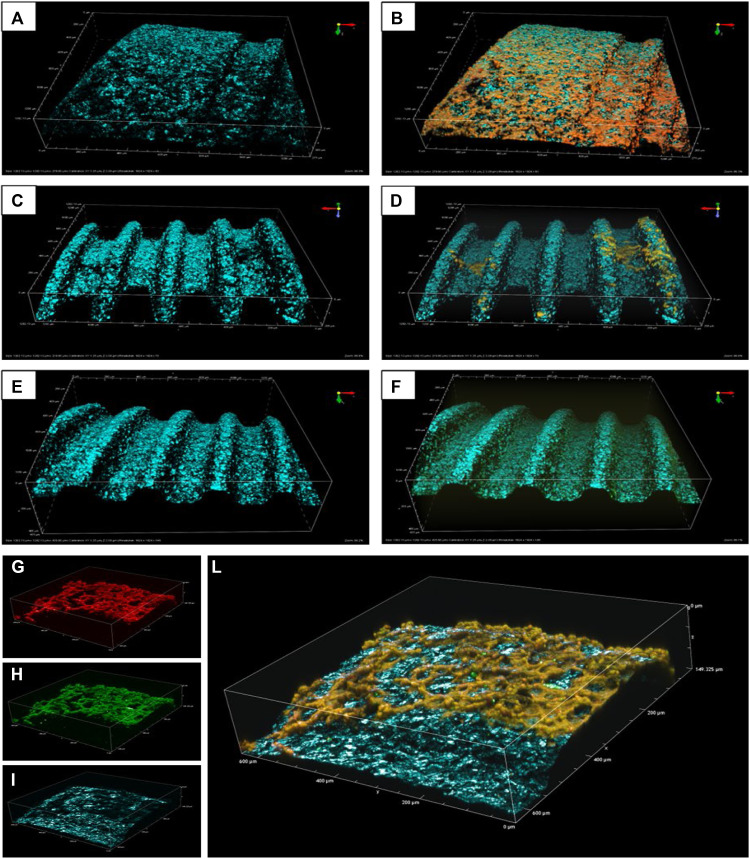
(A–F) CLSM Z-stack images of titanium implant surfaces with attached S. mutans biofilms, acquired using a 10× objective lens and 512-pixel resolution. Reflection mode images are presented in the right column, while the corresponding merged fluorescence–reflection images are shown in the left column. Negative control (untreated) biofilms are displayed in panels (A, B); test samples treated with HIFU show bacterial clusters surrounded by biofilm-free areas (C, D); and positive control samples (Airflow® treated) exhibit a thin, uniform layer of biofilm predominantly located at the thread walls and root junctions (E, F). (G–L): Images of a HIFU-treated sample acquired using a 20× objective lens, showing 3 individual laser channels: 561 nm (g) and 488 nm (h) in fluorescence mode, and 405 nm (i) in reflection mode. The merged fluorescence–reflection image (L) reveals clusters of adhered biofilms surrounded by biofilm-free Ti areas.

The CLSM images of the 3 implants are shown in Fig. 4, Fig. 5. Although the images captured using the 4× objective lens provided a broader view of the titanium implant surface and attached biofilms (Figure 4), they lacked the resolution necessary to discern structural details of either component and offered limited scientific value. Compared to the other optical settings, the 10× objective lens provided an optimal balance between field of view and image detail (Figure 5, A-F). The images captured with this lens displayed both the microscopic morphology of the implant and the attached biofilms. While the 20× objective lens captured only a limited area of 60 × 60 µm—insufficient to encompass even a single implant thread, which measures approximately 80 µm in width in the collar region—it provided high-resolution images of both the biofilms and the titanium surface, offering valuable visual insight into biofilm adherence on the Ti surface (Figure 5, G-L).

The CLSM capturing time was dependent on the objective lens, pixel size, Z stack depth, and Z stack step. As the magnification and pixel size increased, the recommended step size decreased, and the capturing time increased. The time to acquire the images varied between 4 and 60 minutes, depending on these parameters.

### Quantitative results - flow cytometry assays

**Fig. 6:**
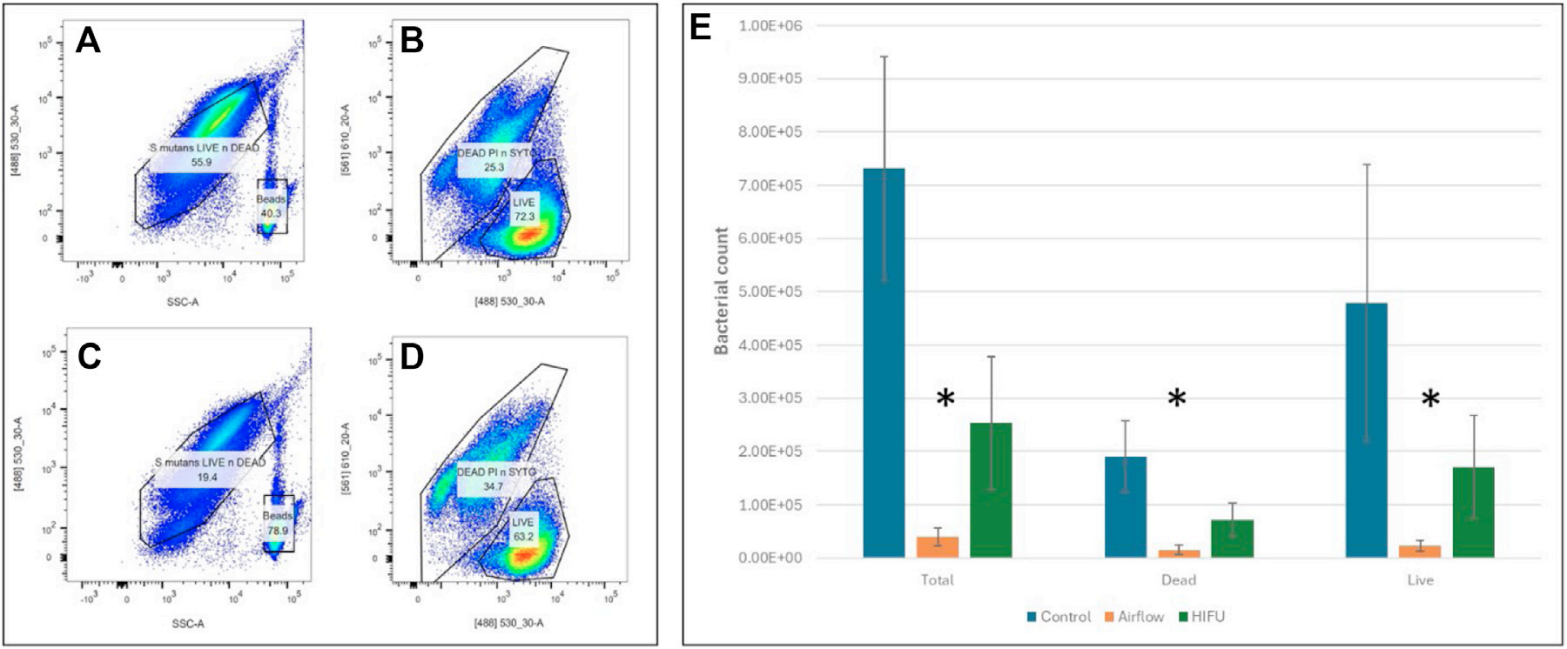
**(left)**: Pseudo colour plots showing the gating structure strategy for total, SYTO9-stained (live), and PI+SYTO9 stained (dead) bacterial populations based on side scatter area (SSC-A) versus the green fluorescence channel [(488)-530/30] and red fluorescence channel [(561)- 610/20]. The upper row (A, B) represents the negative control sample, while the lower row (C, D) shows a HIFU-treated sample. **(right) (E)**: Numerical counts of the 3 bacterial populations for each group are presented. A statistically significant difference (*) was observed between the groups (*p* < .05).

The results of the FCM analysis are presented in Figure 6, demonstrating the predominance of live (SYTO9 stained) bacteria within the biofilms across all sample groups with the average live-to-dead ratio of 2.4:1. The results also showed that both Airflow® and HIFU could significantly debride the attached *S. mutans*.

## Discussion

Although earlier studies have highlighted the potentially therapeutic effects of HIFU on hard dental tissues^61^, ^62^, ^63^, ^64^, ^65^, ^66^, ^67^, ^68^, ^69^, ^70^ and its effectiveness in removing *S. mutans* biofilms from flat titanium surfaces,^49^ the present study aimed to verify whether HIFU produces similar outcomes on bacterial biofilms attached to titanium dental implants. These biofilms have been known to be one of the 2 fundamental factors in the initiation and progression of PI.^6^^,^^7^^,^^19^^,^^66^^,^^71^^,^^72^^,^^73^^,^^74^^,^^75^ Even though there has not been an optimal treatment approach established, the debridement of microbial biofilms attached to implants is the key therapeutic principle.^11^^,^^12^^,^^34^ The principle of debridement was also emphasised in the context of bone tissue regeneration in peri-implantitis, as demonstrated by a systematic review of randomised clinical trials conducted by Castro *et al*. (12).

To achieve this aim, the primary objective of this investigation was to investigate the debridement effect of HIFU on *S. mutans* biofilms attached to Ti dental implants. The combined qualitative and quantitative findings from DCLSM and FCM confirms that HIFU can effectively dislodge *S. mutans* biofilms from titanium implant surfaces, even when a solid barrier separates the transducer from the target.

The debridement capability of HIFU can be explained by the higher calculated maximum negative pressure of 9.9 MPa,^49^ compared to other studies where this value was between 4.1 and 7.6 MPa.^61^ However, the maximum negative pressure was likely not fully transmitted to the biofilms due to attenuation effects, which were compounded by the 4 mm distance between the focal point and the biofilm, as well as the presence of the solid glass barrier.

This attenuation likely explains the reduced debridement efficacy of HIFU relative to Airflow®, in contrast to the findings of Tran et al.,^49^ where both methods demonstrated similar levels of effectiveness. In that study, the biofilms were positioned directly at the transducer’s focal point without any barrier, minimising attenuation and maximising HIFU efficiency. Notably, the presence of a glass barrier highlights a critical observation: HIFU effectively disrupted biofilms without direct contact, demonstrating its ability to transmit acoustic energy through solid interfaces to reach concealed or inaccessible surfaces. These findings provide preliminary *in vitro* evidence supporting further investigation of HIFU for biomedical applications.

Airflow® was selected as the positive control in this investigation due to its established use in clinical applications with well-reported limited efficacy^39^, ^40^, ^41^, ^42^, ^43^, ^44^ (Section 1) and its association with side effects, including life-threatening pneumocephalus^45^, ^46^, ^47^ (Section 1). Considering the unique properties of HIFU, it is plausible that these adverse effects may be mitigated, widening its application to the decontamination phase in the surgical therapies for PI, an indication where Airflow® may be contraindicated.

The dual fluorescence and reflection CLSM technique was first used by Baschong et al. in 2001^54^ to image Ti implant-tissue interfaces and has since seen limited application in dental implant research.^55^ The technique was adapted and integrated into the data collection protocols due to its effectiveness in visualising adherent biofilms and their spatial distribution relative to the titanium surface topography. The use of flow cytometry for distinguishing and counting live and dead bacteria is well-established in microbiological research, including in biofilm studies.^76^ The advantages of FCM in biofilm quantification have been well documented for its rapid and high-throughput analysis, accurate viability assessment, precision, reproducibility, and applicability to complex samples.^77^^,^^78^ However, the combined qualitative imaging DCLSM and FCM have not been extensively explored in dental implant-attached biofilms. The integration of DCLSM imaging with FCM-based bacterial quantification has been validated in our study^49^ and demonstrates potential for application in subsequent research endeavors.

Given the novelty of HIFU in dentistry, there is currently a lack of comparable studies in the literature. However, related research by Vyas et al.^47^^,^^48^ which utilised cavitation generated by oscillating ultrasonic scaler tips, and an investigation by Yamada et al.^49^ which applied cavitation jets, demonstrated the potential of these methods to remove bacterial biofilms.

The surface (being a crucial element in the entire structure of Ti dental implants) plays a pivotal role in molecular interactions and cellular responses leading to osseointegration.^79^, ^80^, ^81^ Consequently various surface roughening and modification methods have been employed to accelerate the process^79^, ^80^, ^81^; However, a systematic review and meta-analysis by Jordana et al.^82^ provides compelling evidence of a correlation between implant surface roughness and the risk of peri-implantitis. This evidence supports the rationale for selecting roughened titanium implants as the experimental substrate in the current study.

The DCLSM images demonstrate unanticipated and pronounced disparities in the debridement patterns elicited by HIFU and Airflow®, consistent with observations from our prior investigation.^49^ HIFU showed the ability to ablate and create areas clear of debris, whereas Airflow appears to "wipe over” the biofilms, leaving behind a mist of residuals. Considering the distinct mechanism of action of HIFU, where acoustic waves induce energy propagation throughout the entire 3-dimensional transmission medium at the microscale, it is reasonable to posit that complete biofilm eradication via HIFU is a plausible outcome.

The limitations of this study include its in vitro design and the restricted availability of the number, types of substrates, and the selection of *S. mutans* as the test bacteria. Although *S. mutans* has not been directly linked to PI,^19^ it is a native inhabitant of the oral cavity, frequently detected in both supra- and subgingival niches,^83^^,^^84^ and is recognised as a pivotal species in biofilm initiation. By November 1, 2024, PubMed indexed 11,488 studies on *S. mutans*, representing 0.78% of all bacteria-related publications and ranking it as the 16th most extensively investigated bacterium.^85^ First described by J. Kilian Clarke in 1924,^86^ *S. mutans* is a Gram-positive, facultative anaerobe within the mutans streptococci group. It is firmly established as a major etiological agent of dental caries and as a critical contributor to oral biofilm initiation and maturation.^22^^,^^84^^,^^87^^,^^88^, ^89^, ^90^, ^91^

This species demonstrates rapid growth, with an exponential rate of 0.24 per hour,^92^ and exhibits distinct pathogenic traits that facilitate its colonisation and promote the development of complex multispecies biofilm communities.^84^ Its biofilm-forming ability is driven by glucan production via glucosyltransferases, glucan-binding proteins, collagen-binding proteins, sucrose-dependent adhesins, and quorum-sensing pathways.^84^^,^^88^ Implant colonisation occurs rapidly, with Fürst et al.^93^ reporting bacterial attachment within 30 minutes of implant placement.^93^ Early colonisers are predominantly Gram-positive aerobes or facultative anaerobes,^90^ among which *S. mutans* displays a strong capacity to initiate biofilm development within the first 16 hours, extending colonisation through salivary pellicle binding.^89^^,^^91^ This pioneer activity facilitates subsequent integration of anaerobic PI-associated pathogens such as *P. gingivalis and F. nucleatum*. As biofilms mature, streptococci reappear as dominant taxa, comprising up to 25% of microbial populations.^87^^,^^89^

Given its central role in biofilm ecology and its ability to form consistent and reproducible biofilms in vitro, this study employed *Streptococcus mutans* solely as a model for early adhesion and biofilm formation, and the results should not be interpreted as directly representative of PI-associated pathogens. Future research should prioritise experiments involving multispecies peri-implantitis-associated biofilms^19^ with particular emphasis on anaerobic pathogens such as *Porphyromonas gingivalis* and *Fusobacterium nucleatum*, within peri-implant pocket model systems. These investigations are essential for advancing the translational potential of HIFU technology toward preclinical animal studies and subsequent human clinical trials. There would be a considerable number of further studies required to convert HIFU into a clinically applicable device from bioengineering perspectives, including device configuration, transmission media, HIFU parameters, debridement effectiveness, and adverse effects, particularly hyperthermia. A handpiece-like HIFU delivery system incorporating a HIFU transducer, immersed in water as both a transmission medium and coolant, has been envisioned as a potential clinically applicable tool and proposed for development in the next phase of research.

## Conclusion

Considering the constraints of this *in vitro* proof-of-concept study on mono-species biofilms, the results indicate that HIFU can effectively remove *S. mutans* biofilms from roughened Ti implant surfaces. Although its overall debridement efficacy was lower than that of Airflow®, HIFU achieved localised zones of complete biofilm eradication and demonstrated the unique ability to penetrate solid barriers. These attributes, combined with its non-invasive nature and absence of residual materials, highlight HIFU's potential as an innovative therapeutic modality for both the prevention and management of peri-implantitis, a growing concern in contemporary implant dentistry, as well as other biofilm-associated infections linked to medical implants. Further research is required to comprehensively elucidate and validate the clinical applicability of this emerging technology.

Moreover, the integration of dual fluorescence–reflection confocal laser scanning microscopy and flow cytometry provided a robust and complementary methodology for the qualitative and quantitative evaluation of biofilms on dental implant surfaces

## Funding

This study was supported by the Australian National Health and Medical Research Council grant (NMHRC, 1188401).

## Author contributions

Study conception and design: Minh Dien Tran, Hien Chi Ngo, Amr Fawzy. Data collection: Minh Dien Tran. Analysis and interpretation of results: Minh Dien Tran, Sheetal Maria Rajan. Alysia Hubbard, Catherine A. Rinaldi. Draft manuscript preparation: Minh Dien Tran, Alysia Hubbard, Catherine A. Rinaldi, Sheetal Maria Rajan, Amr Fawzy. All authors reviewed the results and approved the final version of the manuscript

## Declaration of competing interest

The author is not an Editorial Board Member/Editor-in-Chief/Associate Editor/Guest Editor for this journal and was not involved in the editorial review or the decision to publish this article. The authors declare that they have no known competing financial interests or personal relationships that could have appeared to influence the work reported in this paper.
